# Paternal snus use in puberty and increased risk for asthma and allergies in offspring: a RHINE/RHINESSA two-generation study

**DOI:** 10.1093/ije/dyag035

**Published:** 2026-03-28

**Authors:** Juan Pablo López-Cervantes, Randi J Bertelsen, Vivi Schlünssen, Mathias Holm, Andrei Malinovschi, Lars Modig, Anna Oudin, Francisco Javier Callejas-González, Shyamali C Dharmage, Nils O Jogi, Ane Johannessen, Valentina Lando, Elin Helga Thorarinsdottir, Christer Janson, Simone Accordini, Cecilie Svanes

**Affiliations:** Department of Global Public Health and Primary Care, Centre for International Health, University of Bergen, 5020, Bergen, Norway; Department of Occupational Medicine, Haukeland University Hospital, 5021, Bergen, Norway; Department of Clinical Science, University of Bergen, 5021, Bergen, Norway; Department of Public Health, Research Unit for Environment, Occupation and Health, Danish Ramazzini Centre, Aarhus University, 8000, Aarhus, Denmark; Department of Occupational and Environmental Medicine, School of Public Health and Community Medicine, Institute of Medicine, Sahlgrenska Academy, University of Gothenburg, 41390, Gothenburg, Sweden; Department of Medical Sciences: Clinical Physiology, Uppsala University, 751 85, Uppsala, Sweden; Department of Public Health and Clinical Medicine, Section of Sustainable Health, Umeå University, Umeå, Sweden; Department of Public Health and Clinical Medicine, Section of Sustainable Health, Umeå University, Umeå, Sweden; Department of Respiratory Medicine, Albacete University Hospital Complex, 02008, Albacete, Spain; Allergy and Lung Health Unit, Centre for Epidemiology and Biostatistics, School of Population and Global Health, The University of Melbourne, VIC 3010, Melbourne, Australia; Department of Medical Sciences: Clinical Physiology, Uppsala University, 751 85, Uppsala, Sweden; Department of Global Public Health and Primary Care, University of Bergen, 5020, Bergen, Norway; Unit of Epidemiology and Medical Statistics, Department of Diagnostics and Public Health, University of Verona, 37134, Verona, Italy; Primary Care of the Capital Area, Reykjavik, Iceland and Faculty of Medicine, University of Iceland, Reykjavik, Iceland; Department of Medical Sciences, Respiratory, Allergy and Sleep Research, Uppsala University, 751 85, Uppsala, Sweden; Unit of Epidemiology and Medical Statistics, Department of Diagnostics and Public Health, University of Verona, 37134, Verona, Italy; Department of Global Public Health and Primary Care, Centre for International Health, University of Bergen, 5020, Bergen, Norway; Department of Occupational Medicine, Haukeland University Hospital, 5021, Bergen, Norway

**Keywords:** tobacco, intergenerational, asthma and allergies, asthma epidemiology, epigenetics, surveys and questionnaires

## Abstract

**Background:**

Evidence suggests that a father’s smoking in puberty may adversely impact respiratory health in offspring, possibly through epigenetic changes in germ cells. This study investigates whether snus use starting in or after puberty influences respiratory health in future offspring.

**Methods:**

We analysed Swedish data from RHINE (Respiratory Health in Northern Europe) parents and RHINESSA (Respiratory Health in Northern Europe, Spain and Australia) offspring by using mixed-effect logistic regression to assess the links between paternal snus initiation around puberty and offspring asthma, chronic bronchitis, rhinitis, and eczema, adjusting for paternal and offspring smoking.

**Results:**

We identified 1090 offspring–father pairs. The offspring’s median age was 29 years (17–51) and 55% were women. The maternal line (*n* = 1421) was not analysed, as <1% of mothers used snus in puberty. The offspring of fathers starting snus use in puberty (*n* = 89) had a higher risk of current allergic asthma [odds ratio (OR) 1.42; 95% confidence interval (CI) 1.02–1.97], at least three asthma symptoms with allergy (OR 1.13; 95% CI 1.10–1.21), chronic bronchitis (OR 2.17; 95% CI 1.04–4.54), and eczema (OR 1.45; 95% CI 1.27–1.65). Fathers’ snus use starting after puberty (*n* = 252) showed no consistent associations with offspring outcomes. The effect estimates were consistent after excluding offspring using snus in puberty.

**Conclusion:**

Paternal snus use starting in puberty was associated with a higher risk of asthma and other respiratory and allergic symptoms in offspring. These findings support the hypothesis that exposures in puberty may impact future generations’ respiratory health, possibly through epigenetic changes. This highlights the need for research on exposures during this period and actions to prevent habits that could negatively impact future offspring’s health.

Key Messages

- We studied whether father’s use of snus—a popular moist tobacco product—may influence asthma and allergy in his future offspring, in particular if he started snus use during the susceptible puberty period.
- The offspring of fathers who began using snus in puberty had more symptoms of asthma and allergies than other offspring whose fathers did not use snus or who started using snus after puberty.
- Exposure to snus in puberty, similarly to tobacco smoking, may have potential multigenerational effects, highlighting the importance of addressing tobacco-related habits in youth through public health measures.

## Introduction

Asthma and allergies are common chronic conditions affecting 10%–30% of the population. Asthma is a chronic inflammatory airway disease characterized by variable symptoms and airflow limitation. Allergies commonly manifest as hay fever—nasal mucosa inflammation causing cold-like symptoms—or eczema—an itchy skin reaction [1, 2]. These are multifactorial diseases influenced by exposure to allergens, infections, and immune dysregulation, as well as genetic, epigenetic, and environmental factors. Their aetiology, however, is not fully understood, underscoring the need for further research [1, 3, 4].

Emerging research suggests that chemical and environmental exposures may influence not only the respiratory health of the persons who are directly exposed, but also that of their descendants [5–7]. Epigenetic transfer of information is considered a plausible mechanism through which exposures in one generation may be transmitted to future generations [8]. Moreover, developing germ cells are physiologically more susceptible during stages of reprogramming and differentiation processes [7, 9]. Thus, novel studies suggest that perturbations to epigenetic material in germ cells occurring during vulnerable periods, including puberty, may be passed on and act as drivers of information through generations [10].

A body of evidence suggests that tobacco smoking, particularly during male puberty, may influence respiratory health across generations [5]. Svanes *et al.* found that the offspring of fathers who began smoking in puberty had an increased risk of non-allergic asthma—a pattern not observed in the maternal line [11]. A three-generation causal inference analysis showed similar results, suggesting that paternal puberty-onset smoking may cause asthma in their future offspring [12]. The results are further supported by recent human epigenetic research indicating peripheral blood DNA hypermethylation in offspring related to father’s smoking debut in puberty, at cytosine–phosphate–guanine sites associated with asthma (e.g. cg22402007) and wheeze (e.g. cg11380624; cg10981514) [13]. Regarding nicotine exposure, a mice study showed transgenerational effects of perinatal nicotine exposure in gestating dams transmitted down to the third generation, causing abnormal lung function and changes in asthma phenotype markers [14]. The multigenerational effects of parental smoking are also linked to neurological [15, 16], endocrine [17], and hepatic outcomes [18].

Snus is a high-nicotine-containing product originally derived from tobacco. It has been on the market for many years in Sweden and Norway [19] and is increasingly consumed in other countries, mainly by youth. Recently, nicotine-containing but tobacco-free versions have been introduced to the market [20, 21]. Snus has been linked to respiratory and cardiovascular health effects, including increased risks of asthma, chronic bronchitis, and rhinosinusitis [22], as well as cardiovascular mortality [23]. Our recent analysis of the Respiratory Health in Northern Europe, Spain and Australia study (RHINESSA) showed a three-fold higher asthma risk in women who started snus use in puberty compared with never-users, independently of their smoking habits [24]. Another Scandinavian study found potential effects of snus use *in utero* on early infant lung function [25].

Given the widespread and increasing use of snus among adolescents, its high nicotine content, and emerging evidence of multigenerational effects of nicotine in mice models and of tobacco smoking in human male puberty, it is urgent to examine whether parental snus use among the young could influence respiratory and allergic outcomes in their offspring.

This study aimed to investigate the hypothesis that snus-use initiation during a parent’s puberty may influence the next generation’s risk of asthma and of other respiratory and allergic conditions. To address this, we used unique data from two generations of the linked Respiratory Health in Northern Europe (RHINE) and RHINESSA cohorts. Data from three Swedish study centres were used, as Sweden has a long tradition of snus use and homogeneous tobacco snus products at the time the parent generation in RHINE were born (1944–75) (Table 1).

**Table 1:** Characteristics of the study population by study centre.

Characteristics		Swedish study centres		
	All (*N* = 2511)	Gothenburg (*n* = 624)	Umea (*n* = 880)	Uppsala (*n* = 1007)
Offspring				
Sex (female) (%)	55	52	57	55
Age (years) [median (range)]	30 (17–52)	29 (18–50)	31 (17–52)	2*9* (17–51)
Current asthma^a^				
No current asthma	2224 (88.7)	568 (91.2)	774 (88.0)	882 (87.8)
Current allergic asthma	181 (7.2)	39 (6.3)	64 (7.3)	78 (7.8)
Current non-allergic asthma	103 (4.1)	16 (2.6)	42 (4.8)	45 (4.5)
Current asthma symptoms^b^				
<3 symptoms	2178 (86.7)	553 (88.6)	757 (86.0)	868 (86.2)
≥3 symptoms with allergy	192 (7.7)	43 (6.9)	73 (8.3)	76 (7.6)
≥3 symptoms without allergy	141 (5.6)	28 (4.5)	50 (5.7)	63 (6.3)
Chronic bronchitis^c^	168 (6.7)	40 (6.4)	62 (7.1)	66 (6.6)
Rhinitis^d^	690 (27.7)	167 (27.0)	238 (27.2)	285 (28.7)
Eczema^e^	1169 (46.7)	281 (45.2)	394 (45.0)	494 (49.2)
Parental snus use				
Never snus use	2066 (82.3)	517 (82.9)	693 (78.8)	856 (85.0)
Starting in puberty (≤15 years of age)	97 (3.9)	16 (2.6)	42 (4.8)	39 (3.9)
Starting after puberty (>15 years of age)	348 (13.9)	91 (14.6)	145 (16.5)	112 (11.1)

All values presented as *n* (%) unless otherwise specified.

Missing: >10 missing values for rhinitis (*n* = 23).

a Defined as: use of asthma medication or attack of asthma in the last 12 months with and without rhinitis.

b Defined as: at least three positive answers to eight questions, based on a modified version of the definition provided by Pekkanen *et al.*, 2005: wheeze with breathlessness in the last 12 months; wheeze without cold in the last 12 months; woken by tightness in chest in the last 12 months; woken by attack of shortness of breath in the last 12 months; woken by night cough in the last 12 months; ever had asthma; asthma attack in the last 12 months; currently taking asthma medication. With and without rhinitis.

c Defined as: productive cough almost daily for ≥3 months and/or for ≥2 years. Reference category: No chronic bronchitis.

d Defined as: presence of hay fever/nasal allergies. Reference category: No hay fever/nasal allergies.

e Defined as: ever have had eczema/skin allergy. Reference category: No eczema/skin allergy.

## Methods

### Study population

**Figure 1:**
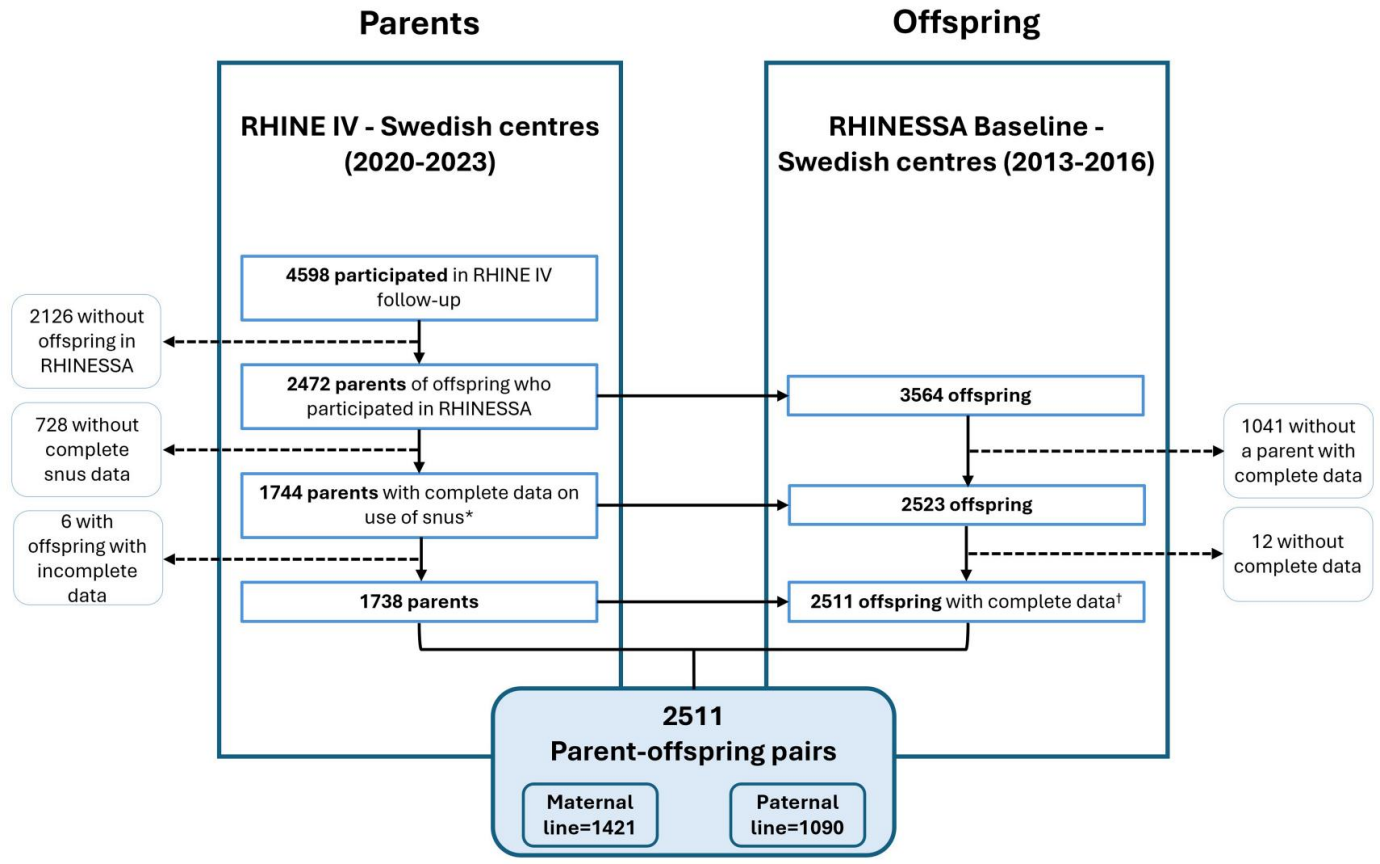
Flowchart of the study sample selection.

This generation study used data from the Swedish study centres of the RHINE study (‘parents’; https://rhine.w.uib.no/ [26]) and the RHINESSA study (‘offspring’; www.rhinessa.net [27]). RHINE is a longitudinal population-based cohort with participants from seven centres in Denmark, Estonia, Iceland, Norway, and Sweden followed via postal questionnaires in four study waves. Using comparable protocols, the RHINESSA study investigates their offspring [27] (Fig. 1). Table 1 shows basic characteristics for 2511 offspring–parent pairs from Gothenburg, Umea, and Uppsala. In the maternal line (*n* = 1421), 7.4% of the offspring had a mother who had ever used snus and only mothers of 0.6% started in puberty; therefore, the maternal line was not studied further.

### Definition of exposure, outcomes, and covariates

Standardized postal/web questionnaires asked about the use of tobacco- and nicotine-containing products (e.g. snus, tobacco smoking), including the time of initiation, intensity, and duration. They also included questions about respiratory health and allergies, as well as general characteristics, other health outcomes, lifestyle, and other exposures.

### Exposure definition—fathers

- Snus use. Paternal snus-use timing was defined based on the responses to questions regarding the regular use of nicotine-containing products that were different from cigarette smoking, the specific use of snus, and the stated age at initiation. Snus use was categorized as: never-use of snus, in puberty (≤15 years of age), and after puberty (>15 years of age).

### Outcome definition—offspring

- Current asthma and asthma symptoms. Current asthma was defined by positive answers to the question: ‘Have you had asthma attacks and/or have you taken asthma medications within the last 12 months?’ Asthma symptoms were assessed by using a symptom score [28] based on the presence (in the past 12 months) of: awoken with chest tightness, awoken with breathlessness, awoken with attack of cough, wheezing with breathlessness, wheezing without having a cold, asthma attacks, and asthma medication. By combining the results with the presence of ‘rhinitis’ (affirmation to ‘Do you have any nasal allergies including hay fever?’), we categorized outcomes into never asthma, current allergic asthma, and current non-allergic asthma; and fewer than three symptoms, at least three symptoms with allergy, and at least three symptoms without allergy.
- Chronic bronchitis. Defined by positive answers to ‘bringing up phlegm’ or ‘having phlegm in the lungs that is difficult to bring up, almost every day for at least three months every year and/or for at least two years in a row’.
- Other symptoms. We included ‘rhinitis’ and ‘eczema’ (‘yes’ to ‘Do you have any nasal allergies including hay fever?’ and ‘Have you ever had eczema or any kind of skin allergy?’, respectively).

### Covariates

**Figure 2:**
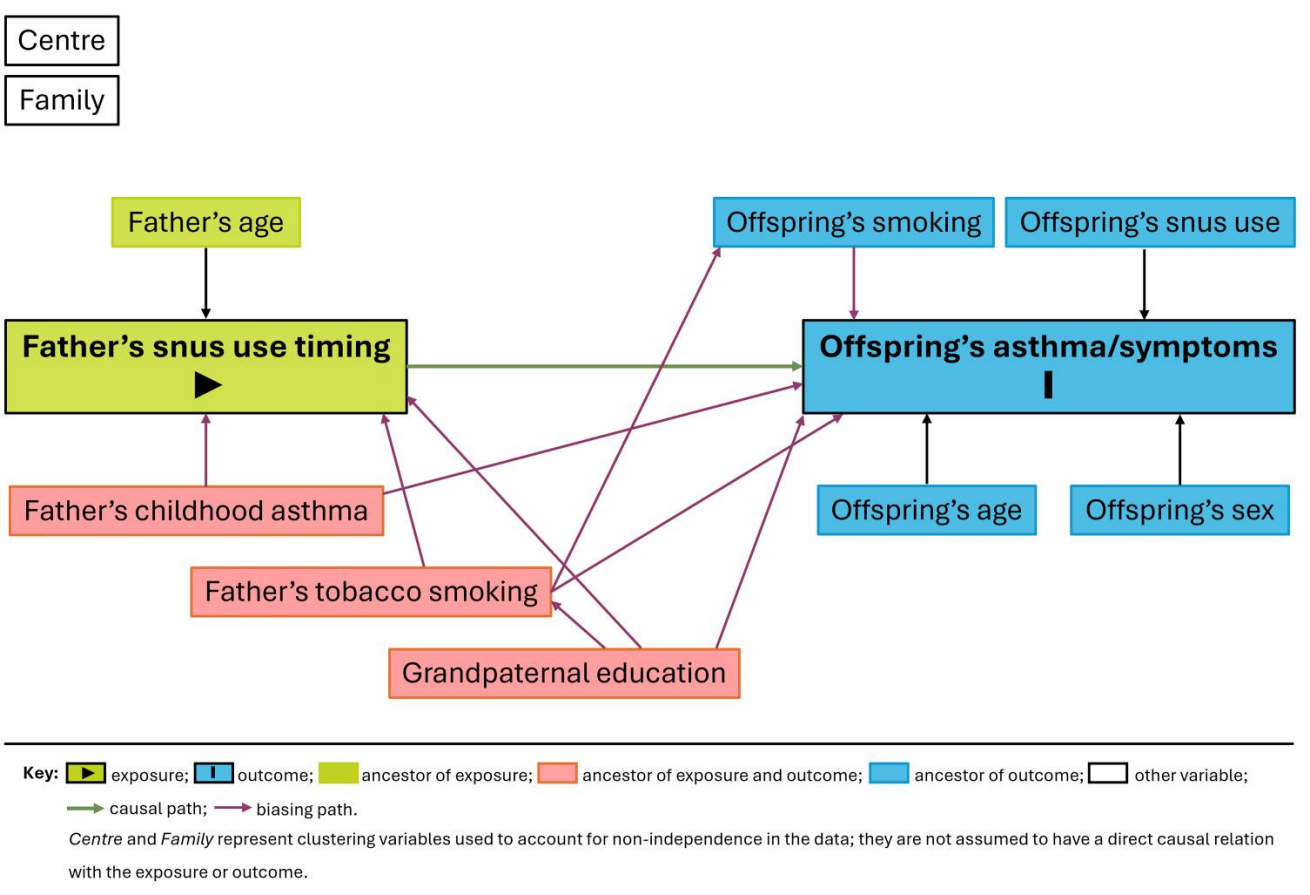
DAG depicting the relationship between the main variables and covariates.

We defined ‘paternal smoking and offspring’s snus use’ as never-use, in puberty (≤15 years of age), and after puberty (>15 years of age). ‘Offspring’s current smoking’ was defined as yes/no. ‘Father’s early- and late-onset asthma’ were defined as having had asthma symptoms at ≤10 or >10 years of age, respectively. We categorized ‘grandpaternal education’ (highest education level attained by any grandparent)—a proxy for socio-economic status—as low (primary/secondary education) and high (college/university education). Confounders were selected by using a directed acyclic graph (DAG; Fig. 2) based on the available scientific evidence, including historical definition with purposeful selection [12].

### Statistical analysis

We included father–offspring pairs with complete information on paternal snus consumption. Baseline characteristics are provided for offspring and fathers. We used mixed-effect multivariate regression models with random intercept to investigate associations of paternal snus use starting in puberty (≤15 years of age) and after puberty (>15 years of age) with offspring’s asthma, asthma symptoms, chronic bronchitis, rhinitis, and eczema. Models accounted for the hierarchical nature of our data: offspring (Level 1) nested within families (Level 2; one father may have several offspring). To account for the intraclass data correlation of families within centres, we present cluster-robust standard errors [12].

All models included the minimum set of confounder and adjusting variables: father’s age, smoking, early- and late-onset asthma [29], grandpaternal education, and offspring’s sex, age, and current smoking [12]. We further examined the effect of the offspring’s own snus use by excluding offspring who reported snus-use initiation at ≤15 years of age, in line with our previous results [24], the effect of paternal allergies, and maternal smoking in pregnancy by excluding offspring with paternal rhinitis and maternal smoking during pregnancy, respectively. Finally, we calculated E-values for the main associations [30] and conducted sensitivity analysis by using Bayesian penalization modelling with default priors to account for sparse data (categories with <10 cases) in the main model, reporting posterior medians for robustness [31, 32]. Analyses were performed by using Stata 18 (StataCorp, College Station, TX, USA).

## Results

### Baseline characteristics

In the paternal line (*n* = 1090), 31% of the offspring had a father who reported using snus, with 8.2% having started in puberty (Table 2). The offspring of fathers who began using snus during puberty were more likely to have a lower education level (54%) compared with those whose fathers never used snus (42%) or started after puberty (38%). Current smoking and snus use in puberty were also more common among offspring whose fathers began using snus in puberty (12.4% and 12.4%, respectively) compared with those whose fathers never used snus (7.5% and 4.7%, respectively) or started after puberty (9.6% and 8.4%, respectively) (Table 3). Mother’s smoking in offspring’s childhood varied little according to the father’s snus use, although there was a tendency toward more maternal smoking if the father started snus use after puberty (Table 2).

**Table 2:** Baseline characteristics of offspring in the paternal line.

	Paternal line			
	All (*N* = 1090)	Never snus (*n* = 749)	Starting in puberty (*n* = 89)	Starting after puberty (*n* = 252)
**Offspring**				
Sex (female) (%)	55	54	55	57
Age (years) [median (range)]	29 (17–51)	29 (18–49)	27 (18–40)	30 (17–51)
Education level				
Low	458 (42.0)	315 (42.1)	48 (53.9)	95 (37.7)
High	629 (57.7)	432 (57.7)	41 (46.1)	156 (61.9)
Unknown	3 (0.3)	2 (0.3)	0 (0.0)	1 (0.4)
Current smoking	91 (8.4)	56 (7.5)	11 (12.4)	24 (9.6)
Snus use				
Never snus use	807 (74.5)	574 (77.1)	64 (71.9)	169 (67.6)
Starting in puberty (≤15 years of age)	67 (6.2)	35 (4.7)	11 (12.4)	21 (8.4)
Starting after puberty (>15 years of age)	210 (19.4)	136 (18.3)	14 (15.7)	60 (24.0)
Maternal smoking^a^				
No	807 (75.1)	564 (76.4)	65 (74.7)	178 (71.5)
Yes	267 (24.9)	174 (23.6)	22 (25.3)	71 (28.5)
**Fathers**				
Age (years) [median (range)]	68 (51–76)	68 (52–76)	64 (51–74)	70 (52–76)
Education level				
Low	347 (31.8)	230 (30.7)	33 (37.1)	84 (33.3)
High	741 (68.0)	518 (69.2)	56 (62.9)	167 (66.3)
Unknown	2 (0.2)	1 (0.1)	0 (0.0)	1 (0.4)
Smoking				
Never smoking	671 (63.5)	498 (69.4)	64 (72.7)	109 (43.6)
Starting in puberty (≤15 years of age)	158 (15.0)	91 (12.7)	20 (22.7)	47 (18.8)
Starting after puberty (>15 years of age)	227 (21.5)	129 (13.0)	4 (4.6)	94 (37.6)
Early-onset asthma^b^				
Never asthma	915 (85.2)	633 (87.1)	70 (79.6))	212 (84.1)
Early-onset asthma	60 (5.6)	42 (5.8)	6 (6.8)	12 (4.8)
Late-onset asthma	92 (8.6)	52 (7.2)	12 (13.6)	28 (11.1)
Rhinitis	259 (24.1)	169 (22.9)	24 (27.3)	66 (26.4)
**Grandparents**				
Education level				
Low	850 (78.0)	568 (75.8)	77 (86.5)	205 (81.4)
High	177 (16.2)	134 (17.9)	8 (9.0)	35 (13.9)
Unknown	63 (5.1)	47 (6.3)	4 (4.5)	12 (4.8)

All values are presented as *n* (%) unless otherwise specified.

Missing values: >10 missing values for maternal smoking (*n* = 16), paternal smoking (*n* = 34), paternal early- and late-onset asthma (*n* = 26), paternal rhinitis (*n* = 15).

a Defined as: mother’s smoking in offspring’s childhood. Answered by offspring in RHINESSA.

b Defined as: ever having asthma and first asthma symptoms at ≤10 or >10 years of age.

**Table 3:** Association between father’s use of snus (never, starting ≤15 years of age ‘in puberty’, starting >15 years of age ‘after puberty’), and offspring’s respiratory and allergic outcomes (*n* = 1090).

	Never snus (*n* = 749)	In puberty (*n* = 89)	After puberty (*n* = 252)	In puberty		After puberty	
	*n* (%)			cOR (95% CI)	aOR^f^ (95% CI)	cOR (95% CI)	aOR^f^ (95% CI)
Current asthma^a^							
Current allergic asthma vs no current asthma	53 (7.1)	9 (10.1)	15 (6.0)	1.52 (0.76–3.03)	1.42 (1.02–1.97)	0.79 (0.65–0.96)	0.70 (0.46–1.10)
Current non-allergic asthma vs no current asthma	35 (4.7)	2 (2.3)	11 (4.4)	0.48 (0.11–2.22)	0.50 (0.10–2.72)	0.92 (0.56–1.50)	0.98 (0.43–2.24)
Current asthma symptoms^b^							
≥3 symptoms with allergy vs <3 symptoms	57 (7.6)	8 (9.0)	16 (6.4)	1.22 (0.70–2.15)	1.14 (1.10–1.21)	0.79 (0.42–1.49)	0.69 (0.28–1.71)
≥3 symptoms without allergy vs <3 symptoms	46 (6.1)	4 (4.5)	18 (7.1)	0.73 (0.10–5.47)	0.73 (0.10–5.70)	1.16 (0.70–1.95)	1.10 (0.53–2.12)
Chronic bronchitis^c^	48 (6.4)	11 (12.4)	19 (7.6)	2.21 (1.21–4.01)	2.17 (1.04–4.54)	1.21 (0.86–1.71)	1.14 (0.61–2.14)
Rhinitis^d^	204 (27.5)	31 (34.8)	71 (28.4)	1.47 (0.87–2.49)	1.41 (0.77–2.58)	1.10 (0.98–1.14)	0.92 (0.83–1.04)
Eczema^e^	354 (47.5)	46 (51.7)	117 (46.4)	1.18 (0.96–1.45)	1.45 (1.27–1.65)	0.97 (0.75–1.25)	0.92 (0.63–1.35)

cOR, crude odds ratio; aOR, adjusted odds ratio; 95% CI, 95% confidence interval.

Missing values: current allergic asthma (*n* = 94); current non-allergic asthma (*n* = 125); ≥3 asthma symptoms with allergy (*n* = 114); ≥3 asthma symptoms without allergy (*n* = 129); chronic bronchitis (*n* = 54); rhinitis (*n* = 59); eczema (*n* = 52).

a Defined as: use of asthma medication or attack of asthma in the last 12 months with and without rhinitis.

b Defined as: at least three positive answers to eight questions, based on a modified version of the definition provided by Pekkanen *et al.*, 2005: wheeze with breathlessness in the last 12 months; wheeze without cold in the last 12 months; woken by tightness in chest in the last 12 months; woken by attack of shortness of breath in the last 12 months; woken by night cough in the last 12 months; ever had asthma; asthma attack in the last 12 months; currently taking asthma medication. With and without rhinitis.

c Defined as: productive cough almost daily for ≥3 months and/or for ≥2 years. Reference category: No chronic bronchitis.

d Defined as: presence of hay fever/nasal allergies. Reference category: No hay fever/nasal allergies.

e Defined as: ever have had eczema/skin allergy. Reference category: No eczema/skin allergy.

f Model adjusted for: paternal smoking; early- and late-onset asthma and age; offspring’s current smoking, age, and sex; and grandpaternal education.

### Paternal snus-use initiation around puberty and offspring’s allergic asthma and symptoms

**Figure 3:**
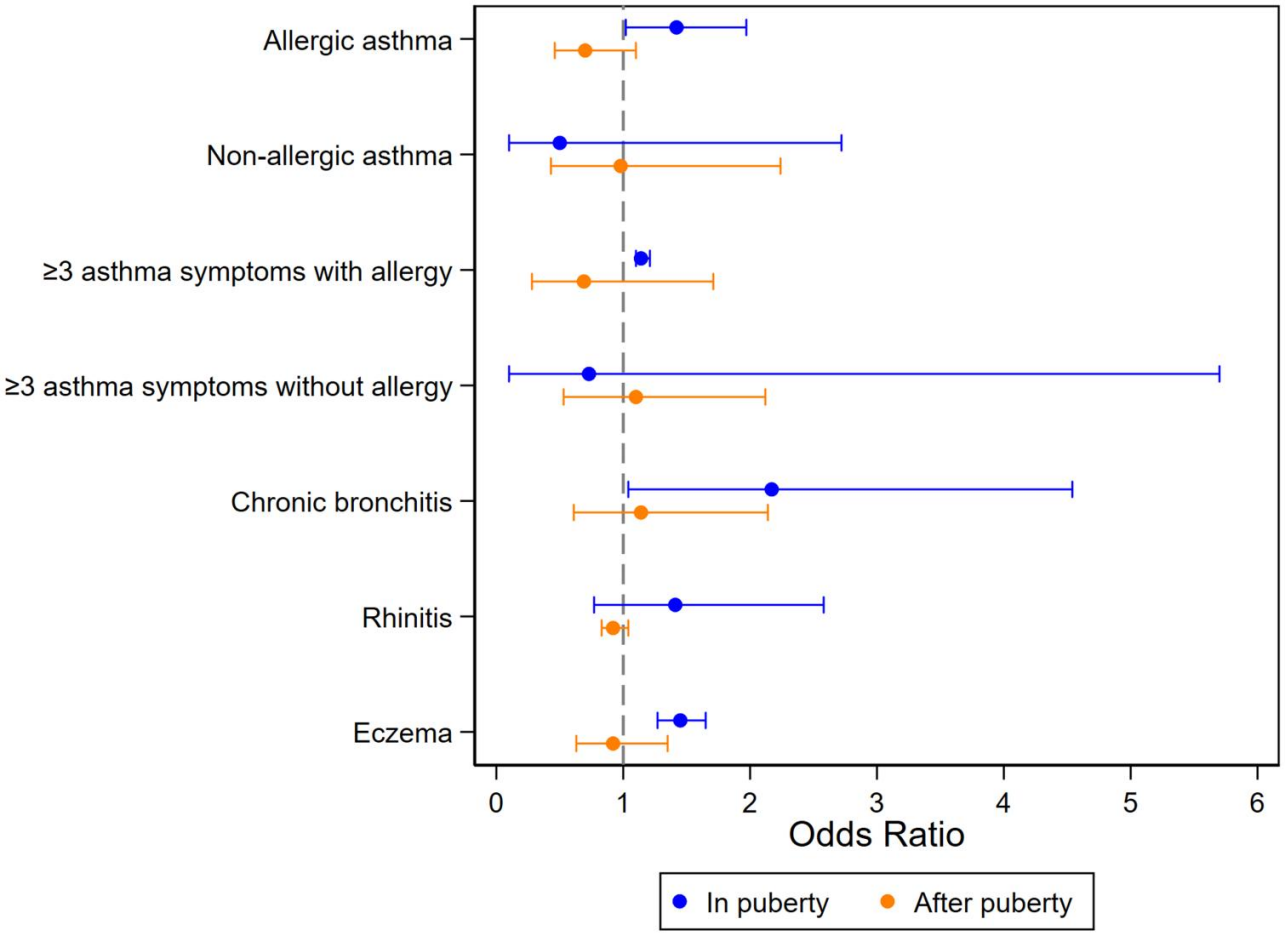
Plot of the associations between paternal snus-use timing and the offspring’s respiratory and allergic outcomes. Footnote: Reference category of the exposure: Never snus use.

After adjustment for paternal smoking and the offspring’s current smoking and other confounding factors, the offspring whose fathers started using snus in puberty had a higher risk of current allergic asthma [odds ratio (OR) 1.42; 95% confidence interval (CI) 1.02–1.97], but not for non-allergic asthma. They also had increased risk of at least three asthma symptoms with allergy (OR 1.14; 95% CI 1.10–1.21), chronic bronchitis (OR 2.17; 95% CI 1.04–4.54), and eczema (OR 1.45; 95%CI 1.27–1.65) (Fig. 3 and Table 3). No consistent risk increase was seen for these outcomes in association with father’s using snus only after puberty (Table 3).

### Sensitivity analyses

Results were consistent after the exclusion of offspring (i) who reported snus use during their puberty (*n* = 67) (Table 4 and Supplementary Table 1), (ii) who used other nicotine-containing products (*n* = 89; estimates not shown), (iii) whose fathers reported rhinitis (*n* = 259) (Table 4 and Supplementary Table 2), or (iv) whose mothers smoked in pregnancy (*n* = 291) (Supplementary Table 4). E-values were highest for main associations with paternal snus use initiated in puberty: chronic bronchitis (3.76; 1.24), eczema (2.26; 1.86), allergic asthma (2.17; 1.16), and at least three asthma symptoms with allergy (1.54; 1.43) (Supplementary Table 3). Following an analysis for sparse data for the outcomes of current allergic asthma and at least three symptoms with allergy, sparse data bias did not attenuate the results (Posterior median OR 1.39; 95% credible intervals 0.9–2.1 and OR 1.19; 95% credible intervals 0.7–2.2, respectively).

**Table 4:** Sensitivity analyses of the association between paternal snus-use initiation starting at ≤15 years of age (in puberty) and offspring’s respiratory and allergic outcomes, (A) in all participants, (B) among offspring who did not start snus use in puberty, and (C) among offspring of fathers without rhinitis.

	Snus-use initiation in puberty		
	A = 1090	B = 1023	C = 831
	aOR (95% CI)		
Allergic asthma^a^	1.42 (1.02–1.97)	1.41 (1.04–1.92)	2.18 (1.54–3.10)
≥3 asthma symptoms with allergy^b^	1.13 (1.10–1.21)	1.11 (0.80–1.55)	1.72 (0.94–3.15)
Chronic bronchitis^c^	2.17 (1.04–4.54)	2.28 (1.10–4.81)	2.10 (1.10–4.01)
Rhinitis^d^	1.41 (0.77–2.58)	1.32 (1.04–1.66)	1.10 (0.78–1.56)
Eczema^e^	1.45 (1.27–1.65)	1.54 (1.10–2.18)	1.26 (0.83–1.91)

a OR, adjusted odds ratio.

All models adjusted for: paternal smoking; early- and late-onset asthma and age; offspring’s current smoking, age, and sex; and grandpaternal education.

Reference exposure category: Never snus.

Missing values: (A) allergic asthma (*n* = 94); ≥3 asthma symptoms with allergy (*n* = 114); chronic bronchitis (*n* = 54); rhinitis (*n* = 59); eczema (*n* = 52). (B) allergic asthma (*n* = 89); ≥3 asthma symptoms with allergy (*n* = 108); chronic bronchitis (*n* = 50); rhinitis (*n* = 54); eczema (*n* = 48). (C) allergic asthma (*n* = 72); ≥3 asthma symptoms with allergy (*n* = 86); chronic bronchitis (*n* = 43); rhinitis (*n* = 47); eczema (*n* = 42).

a Defined as: use of asthma medication or attack of asthma in the last 12 months with rhinitis. Reference category: No current asthma.

b Defined as: at least three positive answers to eight questions, based on a modified version of the definition provided by Pekkanen *et al.*, 2005: wheeze with breathlessness in the last 12 months; wheeze without cold in the last 12 months; woken by tightness in chest in the last 12 months; woken by attack of shortness of breath in the last 12 months; woken by night cough in the last 12 months; ever had asthma; asthma attack in the last 12 months; currently taking asthma medication. With rhinitis. Reference category: <3 symptoms.

c Defined as: productive cough almost daily for ≥3 months and/or for ≥2 years. Reference category: No chronic bronchitis.

d Defined as: presence of hay fever/nasal allergies. Reference category: No hay fever/nasal allergies.

e Defined as: ever have had eczema/skin allergy. Reference category: No eczema/skin allergy.

## Discussion

The potential adverse effects of boys’ snus use in puberty with regard to future offspring’s asthma and allergies were analysed in a two-generation population-based study with 1090 offspring–father units from Sweden. In Sweden, snus has been used for decades, and also when the fathers in our study were young. We found that the offspring of fathers who started using snus in puberty had an increased risk of asthma and allergies compared with those whose fathers started using snus later or never used snus. Results were consistent after adjusting for both fathers’ and offspring’s own tobacco smoking and when excluding offspring who used snus themselves. The association was found particularly for allergic asthma outcomes and eczema, and not for the non-allergic asthma outcomes; the results remained when those with a father with rhinitis were excluded. The difference between early- and later-onset exposure was very consistent in the main analyses and sensitivity analyses, and later-onset snus use was not identified as a risk to offspring health in our study. The risk related to the timing of exposure in the father’s puberty—years before the conception of future offspring, but a time window of critical importance for sperm precursor cell development into mature reproductive cells—is striking.

To our knowledge, no previous human study has examined the effects of snus use in a two-generation scenario (beyond *in utero* exposure) [25]. However, murine and human studies on the multigenerational effects of nicotine and tobacco smoking support our findings [11, 12, 14, 33, 34]. In rat models, nicotine exposure *in utero* (in gestating dams, F0) was found to induce asthma-like changes in offspring (F1) and grand-offspring (F2), and abnormal pulmonary function in great-grand-offspring (F3) [14, 33, 34]. These results imply that nicotine may exert long-lasting effects on respiratory health across generations in mammals, in accordance with our results for the offspring health effects of a father’s exposure to snus—a high-nicotine-content product.

Multigenerational human research on tobacco smoking—another nicotine product—also supports puberty being an age window with higher susceptibility to exposures with regard to effects on the respiratory health of future generations. A father’s tobacco smoking in puberty was linked to a three-fold increased risk of asthma in the offspring—a stronger effect than when fathers started smoking at other time periods or when mothers smoked in pregnancy [11]. These findings were replicated by causal modelling of data from a different study population showing a 43% higher risk of offspring asthma [12] and substantially reduced lung function in offspring [35] related to the father’s puberty-onset smoking. The importance of the preconception origins of respiratory health, specifically of a susceptibility window in parental childhood and puberty, is further supported by studies on exposures as varied as air pollution, overweight, and infections, which were found to be related to offspring respiratory health [5, 6, 36–39]. Thus, our findings on snus align with and extend the results on smoking in humans and nicotine exposure in animal models, as well as with studies of other types of exposures, pointing to an important role for paternal exposures during puberty in shaping future offspring’s respiratory health.

Suggested mechanisms explaining how early parental exposures influence future generations point to epigenetic programming. Recent human epigenetic research has linked blood DNA methylation changes in offspring to father’s smoking initiation in puberty, partly associated with asthma and wheeze [13]. Rehan *et al.* identified specific epigenetic changes in fibroblast markers [fibronectin microRNA (mRNA) and peroxisome proliferator-activated receptor γ] related to asthma phenotype in rat pups following *in utero* nicotine exposure in the great-grandmother [14]. Studies on the paternal line, targeting sperm cells as potential carriers of epigenetic information, have identified the transfer of epigenetic signals in spermatozoa triggered by environmental exposures, including smoking [10, 40, 41]. The timing of exposure in puberty is a challenge in murine models, but Hammer *et al.* showed that preconception smoking in mice led to changes in non-coding mRNA in spermatozoa and in offspring’s phenotype with higher body weight [42]. Overall, current evidence supports that the transfer of epigenetic information via sperm cells may explain the paternal environmental effects on offspring phenotype. Our findings could therefore possibly be explained by paternal snus use during a time window that is critical for sperm precursor cell development into mature reproductive cells, influencing sperm epigenetics and thereby passing on epigenetic information to the offspring. Germ cell development is very different in the male and female lines, but it is important to note that we cannot conclude that this effect is exclusive to the paternal line, given our very limited data on maternal snus exposure in puberty.

Maternal risk factors for offspring asthma, such as maternal smoking in pregnancy, did not affect the consistency of the estimates. While the father’s snus use around conception and pregnancy possibly could influence the mother’s snus/tobacco use, it seems unlikely that it would make a difference whether the father started using snus before or after age 15 years—many years before conception. Crude numbers on mother’s smoking support this (Table 2).

### Strengths and limitations

This innovative paper analyses high-resolution, standardized data from two linked population-based cohorts, exploring, for the first time, the multigenerational effects of a traditional tobacco product that is widely used in the Swedish society. Strong and consistent estimates were observed after accounting for confounding variables and employing statistical methods addressing data hierarchies and clustering. Another strength is the inclusion of multiple outcomes representing a broad spectrum of respiratory and allergic conditions, including a validated symptom asthma score based on the European Community Respiratory Health Survey (ECRHS) [28, 43], all with significance for public health. Temporality between exposure (around the father’s puberty) and outcomes (in the next generation) is clear. Our findings are further supported by robust evidence from mechanistic and epidemiological studies, reinforcing their biological plausibility and consistency.

Our findings may be generalizable to the use of other tobacco- and nicotine-containing products. The existing evidence suggests that the multigenerational influence on respiratory health associated with exposure to tobacco smoking is comparable to that of nicotine exposure; while the effects of each individual exposure need to be further studied, we may infer that the mechanisms underlying the proposed effect on the offspring generation may be relevant also for different types of snus-like products, which are becoming more popular in countries such as Finland and Estonia [21]. Furthermore, we could suspect that this knowledge may be extended to the as yet unexplored effects, on two generations, linked to the use of other products such as e-cigarettes and vapers.

Swedish study centres were chosen because a well-defined, homogeneous snus type was widely used during the father’s youth. Additionally, tobacco snus marketing in Sweden operates within a regulated legal framework. These important factors made confounding and misclassification of exposure assessment less likely compared with data from countries in which tobacco snus is not a traditional product or legally permitted. However, recall bias may have influenced our findings, particularly regarding the timing of snus exposure, which occurred decades before the RHINE IV study collected data from participants who recalled these events. It is unlikely, however, that such bias is related to how the offspring, in a different study, reported their respiratory and allergic health outcomes. Misclassification of exposure would thus be non-differential, potentially leading to underestimation of our findings. Further, misclassification in the offspring’s self-reported health outcomes in a questionnaire-based study is a limiting factor, even if we used internationally standardized ECRHS questionnaires as well as validated disease definitions. However, is unlikely that such misclassification could be related to the father’s snus use, reported in a separate study at a different time. Moreover, residual confounding due to paternal intermittent or dual use of snus and smoking or due to the amount of snus used may also be possible. Finally, unknown confounders cannot be ruled out; however, the E-values suggest that only substantial unknown confounding could explain away the main results [30].

## Conclusions and implications

Our research indicates that paternal snus use starting in puberty, long before the offspring is conceived, may be a risk factor for respiratory and allergic outcomes in the offspring. This supports the overall hypothesis of the preconception origins of health and disease, and the specific hypothesis that exposures during male pubertal development in one generation may impact respiratory health in future generations. In particular, the results highlight the urgency to uncover the potential generational effects of exposures such as the use of nicotine pouches and e-cigarettes, which are rapidly becoming very popular among the younger generations (and eventually future parents). Overall, the presented evidence underscores a need for public health-oriented actions to prevent habits in youth that may harm not only their own future health, but also that of their future offspring.

## Ethics Approval

All Swedish study centres from the RHINE and RHINESSA studies obtained ethical clearance from the corresponding ethical committees (references EPM Sweden 2022–01666-01 and Dnr Uppsala 2013/502, respectively). The participants provided written informed consent prior to participating in each study wave.

## Supplementary Material

dyag035_Supplementary_Data

## Data Availability

Data are available upon reasonable request. Requests for access to data can be made to the RHINE coordinator Professor A.J. (ane.johannessen@uib.no) and to the RHINESSA steering committee PI Professor C.S. (cecilie.svanes@hesle-bergen.no) or vice PI Professor V.S. (vs@ph.au.dk). Reuse of the data must be done in collaboration with the RHINE and RHINESSA study teams. Further information including issues on data security and the sharing of data can be found at www.rhinessa.net. The code used to conduct this analysis can be found at https://github.com/JuanPLopezC/snus_2gen.
