# Supplementary material for: Paternal snus use in puberty and increased risk for asthma and allergies in offspring: a RHINE/RHINESSA two-generation study
Source: Int J Epidemiol. 2026 Mar 28;55(2):dyag035. doi: 10.1093/ije/dyag035 (PMC13032824; doi:10.1093/ije/dyag035)
Supplement: dyag035_Supplementary_Data [file dyag035_supplementary_data.docx]

**Supplementary material**

Paternal snus use in puberty and increased risk for asthma and allergies in offspring. A RHINE/RHINESSA two-generation study
López-Cervantes JP, Bertelsen RJ, Schlünssen V, et al.

**Supplementary figures and tables**

Figure S1. Map with prevalence of ever snus use in parents in study centres of the RHINE study conducted between 2020-2023.

**
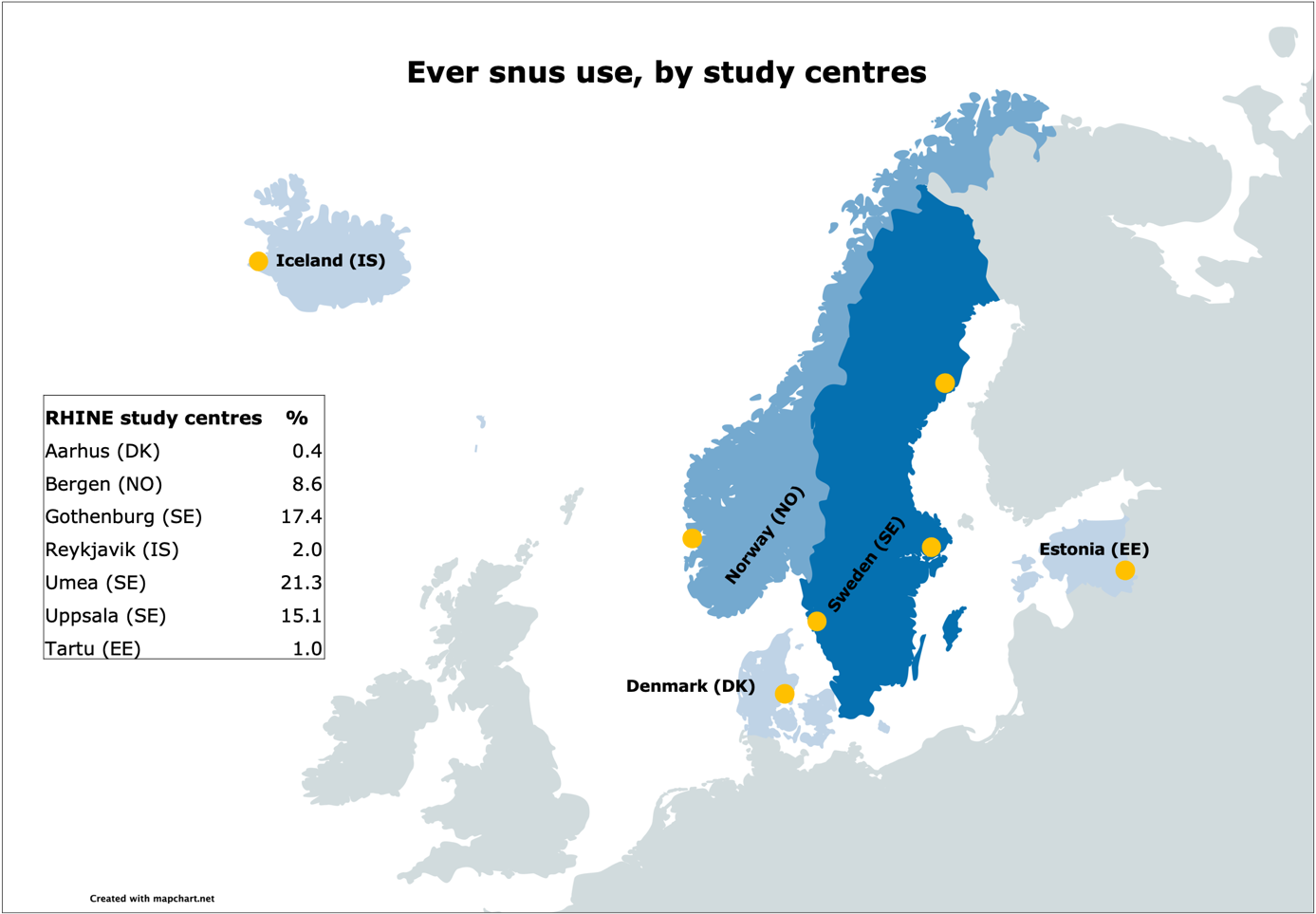
**

Abbreviations: DK (Denmark); NO (Norway); SE (Sweden); IS (Iceland); EE (Estonia)

Table S1. Association between paternal snus use and offspring’s respiratory and allergic outcomes, among offspring who did not start snus use in puberty.

|  | **Paternal line (n=1023)**  ***Snus use initiation*** | | |
| --- | --- | --- | --- |
|  | **Never snus** | **In puberty** | **After puberty** |
| **Current asthma** ^a^ | OR (95% CI) | | |
| Current allergic asthma *vs* no current asthma | 1.00 (ref) | 1.41 (1.04-1.92) | 0.59 (0.43-0.80) |
| Current non-allergic asthma *vs* no current asthma | 1.00 | 0.57 (0.10-3.20) | 1.01 (0.45-2.29) |
| **Current asthma symptoms** ^b^ |  | | |
| ≥3 symptoms with allergy *vs* <3 symptoms | 1.00 | 1.11 (0.80-1.55) | 0.63 (0.31-1.29) |
| ≥3 symptoms without allergy *vs* <3 symptoms | 1.00 | 0.80 (0.11-6.10) | 1.19 (0.59-2.39) |
| **Chronic bronchitis** ^c^ | 1.00 | 2.28 (1.10-4.81) | 0.84 (0.57-1.22) |
| **Rhinitis** ^d^ | 1.00 | 1.32 (1.04-1.66) | 0.93 (0.82-1.10) |
| **Eczema** ^e^ | 1.00 | 1.54 (1.10-2.18) | 0.89 (0.62-1.28) |

**FOOTNOTE:** Model adjusted for: paternal smoking, early- and late-onset asthma and age, offspring´s current smoking, age and sex, and grandparental education.

Missing values: current allergic asthma (n=89); current non-allergic asthma (n=115); ≥3 asthma symptoms (n=108); ≥3 asthma symptoms without allergy (n=118); chronic bronchitis (n=50); rhinitis (n=54); eczema (n=48).

a Defined as: use of asthma medication or attack of asthma in the last 12 months with and without rhinitis.

b Defined as: ≥ three positive answers to eight questions, based on a modified version of the definition provided by Pekkanen et al, 2005: Wheeze with breathlessness in the last 12 months; wheeze without cold in the last 12 months; woken by tightness in chest in the last 12 months; woken by attack of shortness of breath in the last 12 months; woken by night cough in the last 12 months; ever had asthma; asthma attack in the last 12 months; currently taking asthma medication. With and without rhinitis.

c Defined as: productive cough almost daily for at least three months and/or for at least two years. Reference category: No chronic bronchitis.

d Defined as: presence of hay fever/nasal allergies. Reference category: No hay fever/nasal allergies.

e Defined as: ever have had eczema/skin allergy. Reference category: No eczema/skin allergy.

Table S2. Association between paternal snus use and offspring’s respiratory and allergic outcomes, among offspring of fathers without rhinitis.

|  | **Paternal line (n=831)**  ***Snus use initiation*** | | |
| --- | --- | --- | --- |
|  | **Never snus** | **In puberty** | **After puberty** |
| **Current asthma** ^a^ | OR (95% CI) | | |
| Current allergic asthma *vs* no current asthma | 1.00 (ref) | 2.18 (1.54-3.10) | 0.52 (0.23-1.20) |
| Current non-allergic asthma *vs* no current asthma | 1.00 | 0.35 (0.10-1.72) | 0.91 (0.50-1.68) |
| **Current asthma symptoms** ^b^ |  | | |
| ≥3 symptoms with allergy *vs* <3 symptoms | 1.00 | 1.72 (0.94-3.15) | 0.47 (0.33-0.68) |
| ≥3 symptoms without allergy *vs* <3 symptoms | 1.00 | 0.47 (0.12-1.83) | 0.86 (0.49-1.50) |
| **Chronic bronchitis** ^c^ | 1.00 | 2.10 (1.10-4.01) | 1.45 (0.82-2.55) |
| **Rhinitis** ^d^ | 1.00 | 1.10 (0.78-1.56) | 0.86 (0.66-1.12) |
| **Eczema** ^e^ | 1.00 | 1.26 (0.83-1.91) | 0.96 (0.60-1.52) |

**FOOTNOTE:** Model adjusted for: paternal smoking, early- and late-onset asthma and age, offspring´s current smoking, age and sex, and grandparental education.

Missing values: current allergic asthma (n=72); current non-allergic asthma (n=117); ≥3 asthma symptoms with allergy (n=86); ≥3 asthma symptoms without allergy (n=96); chronic bronchitis (n=43); rhinitis (n=47); eczema (n=42).

a Defined as: use of asthma medication or attack of asthma in the last 12 months with and without rhinitis.

b Defined as: ≥ three positive answers to eight questions, based on a modified version of the definition provided by Pekkanen et al, 2005: Wheeze with breathlessness in the last 12 months; wheeze without cold in the last 12 months; woken by tightness in chest in the last 12 months; woken by attack of shortness of breath in the last 12 months; woken by night cough in the last 12 months; ever had asthma; asthma attack in the last 12 months; currently taking asthma medication. With and without rhinitis.

c Defined as: productive cough almost daily for at least three months and/or for at least two years. Reference category: No chronic bronchitis.

d Defined as: presence of hay fever/nasal allergies. Reference category: No hay fever/nasal allergies.

e Defined as: ever have had eczema/skin allergy. Reference category: No eczema/skin allergy.

Table S3. E-values of the main associations between paternal snus use and offspring’s respiratory and allergic outcomes.

|  | **Paternal line (n=1090)**  ***Snus use initiation*** | | |
| --- | --- | --- | --- |
|  | **Never snus (n=749)** | **In puberty (n=89)** | **After puberty (n=252)** |
| **Current asthma** ^a^ | E (CI) ^f^ | | |
| Current allergic asthma *vs* no current asthma | Reference | 2.17 (1.16) | 2.21 (1.00) |
| Current non-allergic asthma *vs* no current asthma |  | 3.41 (1.00) | 1.17 (1.00) |
| **Current asthma symptoms** ^b^ |  | | |
| ≥3 symptoms with allergy *vs* <3 symptoms |  | 1.54 (1.43) | 2.26 (1.00) |
| ≥3 symptoms without allergy *vs* <3 symptoms |  | 2.10 (1.00) | 1.43 (1.00) |
| **Chronic bronchitis** ^c^ |  | 3.76 (1.24) | 1.54 (1.00) |
| **Rhinitis** ^d^ |  | 2.17 (1.00) | 1.39 (1.00) |
| **Eczema** ^e^ |  | 2.26 (1.86) | 1.39 (1.00) |

**FOOTNOTE:** Abbreviations: E-value (E), confidence interval (CI).

Calculated from main model (Table 3) adjusted for: parental smoking, early- and late-onset asthma and age, offspring´s current smoking, age and sex, and grandparental education.

Missing values: current allergic asthma (n=94); current non-allergic asthma (n=125); ≥3 asthma symptoms with allergy (n=114); ≥3 asthma symptoms without allergy (n=129); chronic bronchitis (n=54); rhinitis (n=59); eczema (n=52).

a Defined as: use of asthma medication or attack of asthma in the last 12 months with and without rhinitis.

b Defined as: ≥ three positive answers to eight questions, based on a modified version of the definition provided by Pekkanen et al, 2005: Wheeze with breathlessness in the last 12 months; wheeze without cold in the last 12 months; woken by tightness in chest in the last 12 months; woken by attack of shortness of breath in the last 12 months; woken by night cough in the last 12 months; ever had asthma; asthma attack in the last 12 months; currently taking asthma medication. With and without rhinitis.

c Defined as: productive cough almost daily for at least three months and/or for at least two years. Reference category: No chronic bronchitis.

d Defined as: presence of hay fever/nasal allergies. Reference category: No hay fever/nasal allergies.

e Defined as: ever have had eczema/skin allergy. Reference category: No eczema/skin allergy.

f Lower limit (LL) of the CI is indicated in brackets

Table S4. Association between paternal snus use and offspring’s respiratory and allergic outcomes, among offspring of mothers who did not smoke in their pregnancy.

|  | **Paternal line (n=799)**  ***Snus use initiation*** | | |
| --- | --- | --- | --- |
|  | **Never snus** | **In puberty** | **After puberty** |
| **Current asthma** ^a^ | OR (95% CI) | | |
| Current allergic asthma *vs* no current asthma | 1.00 (ref) | 1.95 (0.90-4.23) | 0.85 (0.47-1.51) |
| Current non-allergic asthma *vs* no current asthma | 1.00 | 0.64 (0.11-3.81) | 1.00 (0.41-2.46) |
| **Current asthma symptoms** ^b^ |  | | |
| ≥3 symptoms with allergy *vs* <3 symptoms | 1.00 | 1.45 (0.86-2.47) | 0.83 (0.56-1.23) |
| ≥3 symptoms without allergy *vs* <3 symptoms | 1.00 | 1.12 (0.14-9.10) | 1.14 (0.50-2.62) |
| **Chronic bronchitis** ^c^ | 1.00 | 3.01 (0.58-15.6) | 1.56 (0.54-4.53) |
| **Rhinitis** ^d^ | 1.00 | 1.18 (0.74-1.89) | 0.92 (0.56-1.51) |
| **Eczema** ^e^ | 1.00 | 1.48 (1.03-2.13) | 1.00 (0.60-1.68) |

**FOOTNOTE:** Model adjusted for: paternal smoking, early- and late-onset asthma and age, offspring´s current smoking, age and sex, and grandparental education.

Missing values: current allergic asthma (n=70); current non-allergic asthma (n=87); ≥3 asthma symptoms with allergy (n=79); ≥3 asthma symptoms without allergy (n=101); chronic bronchitis (n=36); rhinitis (n=40); eczema (n=35).

a Defined as: use of asthma medication or attack of asthma in the last 12 months with and without rhinitis.

b Defined as: ≥ three positive answers to eight questions, based on a modified version of the definition provided by Pekkanen et al, 2005: Wheeze with breathlessness in the last 12 months; wheeze without cold in the last 12 months; woken by tightness in chest in the last 12 months; woken by attack of shortness of breath in the last 12 months; woken by night cough in the last 12 months; ever had asthma; asthma attack in the last 12 months; currently taking asthma medication. With and without rhinitis.

c Defined as: productive cough almost daily for at least three months and/or for at least two years. Reference category: No chronic bronchitis.

d Defined as: presence of hay fever/nasal allergies. Reference category: No hay fever/nasal allergies.

e Defined as: ever have had eczema/skin allergy. Reference category: No eczema/skin allergy.
